# Incidence of Non-Simultaneous Contralateral Second Hip Fractures: A Single-Center Irish Study

**DOI:** 10.7759/cureus.11154

**Published:** 2020-10-25

**Authors:** Patrick Nolan, Lauren Tiedt, Prasad Ellanti, Tom McCarthy, Niall Hogan

**Affiliations:** 1 Trauma and Orthopaedics, St. James' Hospital, Dublin, IRL

**Keywords:** second hip fracture, incidence, bilateral hip fractures, secondary prevention

## Abstract

Introduction

Hip fractures are a significant cause of morbidity and mortality in the elderly and are also associated with increased healthcare costs. A second contralateral hip fracture can lead to even more complications and healthcare costs. A significant proportion of the Irish hip fracture population does not receive a bone health assessment or falls specialist assessment to reduce the risk of future falls and fractures. This study aimed to analyze the incidence of a non-simultaneous contralateral hip fracture in an Irish population.

Methods

We retrospectively analyzed 1,344 patients presenting to our institution with a hip fracture from January 2007 to June 2019. Patients aged ≥ 60 years old presenting with a neck of femur or pertrochanteric fracture were included in our study. We excluded patients who had sub-trochanteric and femoral shaft fractures, high energy fractures, and pathological fractures. We also excluded patients less than 60 years old, as fractures in these younger patients may not be purely related to osteoporosis.

Results

A total of 1,099 hip fractures meeting the inclusion criteria were treated at our unit during the designated time period. A total of 102 (9.3%) patients experienced a second hip fracture. The mean age at first presentation in our institution was 78.5 years old, with a mean time between first and second hip fractures of 37.2 months.

Conclusions

Patients presenting with a second hip fracture may represent 9.3% of the Irish hip fracture population. We hope that this study will help inform on the rate of second hip fractures in an Irish population and help advocate for improved resources and implementation of secondary prevention strategies.

## Introduction

Hip fractures are a significant cause of morbidity and mortality in the elderly. Globally, the number of hip fractures in 2010 was estimated to be approximately 2.7 million cases per year [1]. However, conservative estimates predict this number to increase to 4.5 million by 2050 [1].

The Irish Hip Fracture Database (IHFD) recorded 3,750 hip fractures in Ireland in 2018 [2]. These fractures are associated with significant healthcare costs. It was estimated to cost the Irish health service approximately €45 million in 2018 [2]. The financial cost associated with hip fractures in Ireland is estimated to increase to approximately €162 million per year by 2046 [3].

The Central Statistics Office (CSO) in Ireland predicts that the population over the age of 65 is set to increase from 629,800 in 2016 to nearly 1.6 million in 2051 [4]. The increasing life expectancy and aging population are set to significantly increase the number of patients presenting with hip fractures to Irish hospitals. This is going to present significant challenges to the Irish healthcare system.

In acknowledgment of the increasing numbers of hip fractures, greater emphasis has been placed on secondary prevention of hip and other fragility fractures in the elderly Irish population. One of the primary objectives of the IHFD audit is to ensure that all hip fracture patients receive a bone health assessment and a falls specialist assessment to reduce the risk of further falls and fragility fractures [2]. However, in 2018, these figures were only at 84% and 70%, respectively [2]. The Fragility Fracture Network, which is a global organization, advocate for improvements in secondary prevention through a systematic, multi-disciplinary approach to fragility fracture care with the aim of restoring function and preventing subsequent fractures without delay [1].

Despite these secondary prevention efforts, patients continue to present to the hospital with a second hip fracture. The incidence of non-simultaneous bilateral hip fractures varies throughout the literature and ranges from 1.7% to 15% [5-11]. The contralateral second hip fracture rate for the Irish population is currently not known. Second hip fractures have been associated with increased mortality and significantly increased costs [12,13]. Therefore, there is strong motivation to implement effective secondary prevention strategies in these patients.

The objective of this study is to establish the incidence of non-simultaneous bilateral hip fractures in an Irish population including the demographics and fracture type symmetry. We hope that these data can help accurately estimate the number of patients suffering from second hip fractures in an Irish population and help advocate for improved resources and implementation of secondary prevention strategies.

## Materials and methods

We retrospectively analyzed 1,344 patients who presented to our institution with a hip fracture from January 2007 to June 2019. This population was obtained from the Hospital In-Patient Enquiry (HIPE) department, which collects data on all patients presenting with hip fractures in our institution.

All patients who sustained a neck of femur or a pertrochanteric hip fracture were included in our study. We excluded patients who had sub-trochanteric and femoral shaft fractures, high energy fractures, and pathological fractures. We also excluded patients less than 60 years old, as fractures in these younger patients may not be purely related to osteoporosis.

The patients' radiographs were then analyzed using the National Integrated Medical Imaging System (NIMIS)/Picture Archiving System (PACS) to establish the demographic data, type of fracture, and surgical treatment. Additionally, in patients with a previous contralateral hip fracture, we noted the age at initial fracture, type of fracture, and time in months between the two fractures.

## Results

A total of 1,099 hip fractures meeting the inclusion criteria were treated at our unit during the designated time period. The study included 794 females (72%) and 305 (28%) males, with a mean age of 79.7 years (Table 1). The fracture was a femoral neck fracture in 861 (78%) cases and a pertrochanteric fracture in 238 (22%) cases. The fracture was on the right side in 528 (48%) cases and on the left side in 571 (52%) cases.

**Table 1:** Patient demographics including sex, age, side of hip fracture, and fracture type for all hip fractures, first hip fracture, and second hip fracture.

Patient Demographics	All Hip Fractures, n (%)	First Hip Fracture, n (%)	Second Hip Fracture, n (%)
Number	1099 (100)	997 (90.7)	102 (9.3)
Sex			
Female	794 (72)	713 (64.9)	81 (7.4)
Male	305 (28)	284 (25.8)	21 (1.9)
Mean age	79.7	79.4	81.7
Side			
Right	528 (48)	479 (43.6)	49 (4.5)
Left	571 (52)	518 (47.1)	53 (4.8)
Fracture type			
Femoral neck	861 (78)	795 (72.3)	66 (6.0)
Pertrochanteric	238 (22)	202 (18.4)	36 (3.3)

Neck of femur fractures

The patients with neck of femur fractures constituted a significant majority in this series of patients (861 cases or 78%). Of these, 772 cases were treated with an arthroplasty procedure. A hemiarthroplasty was the treatment of choice in 756 (87.8% of all neck of femur fractures) cases and a total hip arthroplasty in a further 16 (1.9%) cases.

A total of 72 patients underwent internal fixation for their neck of femur fractures. Dynamic hip screw (DHS) was the fixation of choice in 63 (7.3%) cases, whereas cannulated screw fixation was performed in 9 (1%) cases.

A non-operative course was pursued in 17 (2%) patients either due to patient preference or unsuitability for anesthesia.

Pertrochanteric fractures

Of the 238 patients with a pertrochanteric fracture, 174 (73.1%) were treated with a DHS as the fixation of choice and 58 (24.4%) were treated with a cephalomedullary nail. A further six patients were treated non-operatively due to patient preference or unsuitability for anesthesia (2.5%).

Contralateral second hip fractures

A total of 102 (9.3%) patients of the 1,099 included patients had already been treated for a contralateral hip fracture. A total of 81 (79.4%) of these cases were females and 21 (20.6%) were males. The mean age ± standard deviation at the time of first fracture in this cohort was 78.5 ± 8.0 years and that for age at the time of second hip fracture 81.7 ± 8.6 years (Table 2). The breakdown between men and women for age at the time of first and second hip fractures is given in Table 2.

**Table 2:** Patient’s age at first hip fracture, age at second fracture, and time between hip fractures (mean ± standard deviation).

Patient Group	Age at First Hip Fracture (Years)	Age at Second Hip Fracture (Years)	Time between Fractures (Months)
Women	78.6 ± 8.0	81.9 ± 8.4	36.4 ± 40.7
Men	78.2 ± 8.8	80.3 ± 9.5	41.0 ± 32.8
All	78.5 ± 8.0	81.7 ± 8.6	37.2 ± 39.1

The mean time ± standard deviation from first hip fracture to second hip fracture in this population was 37.2 ± 39.1 months overall, with 36.4 ± 40.7 and 41.0 ± 32.8 months for women and men, respectively (Table 2).

A large proportion of the second hip fractures (23.5%) occurred within the first year following the initial hip fracture in our study. More than half of these fractures (55%) occurred within the first five years of the initial hip fracture. The figure for year 2 was 6.9% and that for years 3, 4, and 5 was 7.8% each. The second hip fracture occurred in year 6 in 5.9% of cases, with the numbers remaining low in the subsequent years.

In this population, 76 (74.5%) patients presented with the same fracture pattern on both occasions (Table 3). A total of 56 (54.9%) of these cases were femoral neck fractures on both occasions and 20 (19.6%) cases were pertrochanteric fractures on both occasions. However, 26 (25.5%) cases presented with an asymmetric hip fracture pattern.

**Table 3:** Number of patients with different first and second hip fracture patterns in patients presenting with a second contralateral hip fracture.

First and Second Hip Fracture Types	Number of Patients
Femoral neck fracture both times	56
Pertrochanteric fracture both times	20
Femoral neck and then pertrochanteric fracture	10
Pertrochanteric and then femoral neck fracture	16

The patients with a neck of femur fracture also constituted a majority of the second contralateral hip fracture cohort (72 cases or 70.6%). A hemiarthroplasty was the treatment of choice in 68 (94.4% of all neck of femur fractures in this population) cases. A total of three patients underwent internal fixation for their neck of femur fracture. DHS was the fixation of choice in two (2.8%) patients, whereas one (1.4%) patient underwent cannulated screw fixation. Only one (1%) patient was treated non-operatively in this cohort due to unsuitability for anesthesia.

Of the 30 pertrochanteric fractures, 22 (73.3%) were treated with a DHS as the fixation of choice and 8 (26.7%) were treated with a cephalomedullary nail.

## Discussion

In our study, the incidence of non-simultaneous contralateral hip fracture is 9.3%. This figure is comparable to the incidence reported in the literature, which ranges from 1.7% to 15% [5-11]. Mazzucchelli et al. report an annual incidence of second hip fracture among patients with a previous hip fracture as 956 per 100,000 population [7]. The Irish population over the age of 65 is forecasted to increase from 629,800 in 2016 to nearly 1.6 million in 2046 [4]. With this aging population, the number of patients presenting to Irish hospitals with both first and second hip fractures is expected to increase considerably.

In total, there were 102 patients who presented to our institution with a second hip fracture, of which 81 (79.4%) were females and 21 (20.6%) were males. This suggests that females may have an increased risk of second hip fracture when considering the sex ratio for all hip fractures (72% female vs 28% male). There have been several studies that have looked at risk factors for a second hip fracture. Liu et al. performed a meta-analysis analyzing the risk factors for second contralateral hip fracture in the elderly population. They concluded that female sex, nursing home residents, osteoporosis, visual impairment, cognitive impairment, dizziness, and cardiac and respiratory conditions were all associated with an increased risk for a second hip fracture [14].

This study found that 74.5% of the contralateral hip fractures were of the same fracture type as the initial hip fracture (54.9% femoral neck fractures vs 19.6 pertrochanteric fractures). This figure is consistent with the available literature, which reports a figure ranging from 64% to 83% [15]. The reasons for different fracture patterns are not clearly understood and different explanations have been postulated for both fracture symmetry and asymmetry. Shabat et al. explain that fracture symmetry can be attributed to the fact that each patient possesses their own unique gait and bone architecture which results in the same type of fall ultimately resulting in the same type of fracture [5].

Our study found that 25.5% of patients had an asymmetric fracture type (9.8% had a femoral neck fracture followed by a pertrochanteric fracture vs 15.7% who had a pertrochanteric fracture followed by a femoral neck fracture). One possible explanation put forth for this asymmetric fracture pattern is that there is an increased incidence of a trochanteric fracture with increasing age in hip fracture patients [15]. However, in our study, 16 patients presented with a pertrochanteric hip fracture followed by a femoral neck fracture in comparison to 10 patients who presented with a femoral neck fracture followed by a pertrochanteric fracture. Therefore, further research is required to adequately explain the reasons for an asymmetric hip fracture pattern in this population.

The mean time (± standard deviation) in our study for all patients to the second hip fracture was 37.2 months (± 39.1). The mean time was noted to be 41.0 ± 32.8 months vs 36.4 ± 40.7 months for men and women, respectively. Figure 1 in our study demonstrates that for our cohort the risk of a second hip fracture is highest in the first year, with most of the remaining fractures occurring within five years of the initial hip fracture. These findings are supported by the literature where several studies have demonstrated that most contralateral hip fractures will occur within the first 48 months following an initial hip fracture [8]. However, Sobolev et al. demonstrated that the risk of a contralateral hip fracture persists for at least 10 years following the initial hip fracture [10].

The morbidity and mortality associated with hip fractures are high and are associated with significant healthcare costs. Therefore, great emphasis has been placed on secondary prevention of hip fractures and other fragility fractures. The IFHD was established in 2012 as international evidence has shown us that established standards of care, regular audit, and feedback have demonstrated measurable improvements in hip fracture outcomes including mortality and healthcare costs [16]. Two of these standards of care include a bone health assessment and a multidisciplinary falls assessment to reduce the risk of future falls and fractures [2]. There have been significant improvements in these figures from 2013 to 2018 (47% vs 84% of patients received a bone health assessment and 64% vs 70% of patients received a multidisciplinary falls assessment) [2,16]. These figures demonstrate that there is a better recognition of the importance of secondary prevention in these patients. However, further improvements are needed in the Irish hip fracture population with regard to secondary prevention to meet best practice care.

There are several limitations to our study. This was a retrospective study based on prospectively collected data. There is a possibility that some hip fractures were missed or that data were recorded erroneously. Our institution is a large university hospital in Dublin, with several other hospitals in close proximity that manage hip fracture patients. Some patients who were managed in our center with an initial hip fracture may have then subsequently presented to a different center with a contralateral hip fracture. This cohort will not have been captured in our study and may mean that the incidence of second hip fractures may be higher than estimated in our study.

## Conclusions

Hip fractures are an important public health problem with numbers predicted to increase dramatically as the population ages. The number of patients presenting with a second hip fracture is also set to increase. In an Irish population in a single center, our study estimates the frequency of a second hip fracture as 9.3%, with 74.5% of fractures being identical to the first fracture. The average time to a second hip fracture in our study was estimated at 37.2 months. We hope that this study will help to accurately estimate the incidence of a second hip fracture among an Irish population and help advocate for continued improvements in the secondary prevention of hip fractures.
